# A new species of the genus *Metaphire* (Oligochaeta, Megascolecidae) from northern China with data from the mitochondrial genome

**DOI:** 10.3897/zookeys.1278.182854

**Published:** 2026-04-22

**Authors:** Qingyu Liu, Huifeng Zhao, Nonillon M. Aspe, Yufeng Zhang, Wenqi Zhang, Zhenjiang Zhang, Yanjie Dong

**Affiliations:** 1 College of Life Science, Shenyang Normal University, Shenyang 110034, Liaoning, China College of Environment and Life Sciences, Mindanao State University at Naawan Naawan Philippines; 2 Hebei Key Laboratory of Animal Diversity, College of Life Science, Langfang Normal University, Langfang 065000, Hebei, China College of Life Science, Shenyang Normal University Shenyang China; 3 College of Environment and Life Sciences, Mindanao State University at Naawan, Naawan 9023, Misamis Oriental, Philippines College of Life Science, Langfang Normal University Langfang China; 4 Langfang Ecological Environment Monitoring Center of Hebei Province, Langfang 065000, Hebei, China Langfang Ecological Environment Monitoring Center of Hebei Province Langfang China

**Keywords:** Annelida, ASAP, COX1, East Asia, GMYC, taxonomy

## Abstract

This study describes a new species, *Metaphire wenanensis* Liu & Zhao, **sp. nov**. integrating morphological characters and molecular data. The new species is assigned to the *M. houlleti* group, and is characterized by three pairs of spermathecal pores at 6/7–8/9, and absence of genital markings both anterior and posterior to the clitellum. The spermathecae are connected to the body wall through a disc-shaped atrium, which is interposed between them and the body wall. The molecular phylogenetic position of *M. wenanensis* Liu & Zhao, **sp. nov**. within the *M. houlleti* group was analyzed using mitochondrial genome data. This marks the initial documentation of a new species within the family Megascolecidae in northern China since the last decades.

## Introduction

Earthworms constitute a substantial proportion of macrofaunal biomass and are key components of soil fauna communities across most habitats (Shukla et al. 2024), playing pivotal roles in soil biological systems. As critical drivers of soil ecosystems, research into their classification and phylogeny not only enhances our understanding of their ecological functions but also carries profound significance for biodiversity conservation.

Currently, there are more than 6,000 recorded species of earthworms around the whole world, primarily distributed in terrestrial ecosystems (Jiang and Qiu 2024). Megascolecidae Rosa, 1891 is one of the largest families within the class Oligochaeta (Sun 2013). The family contains 85 genera, among which the genera *Amynthas* Kinberg, 1867 and *Metaphire* Sims & Easton, 1972 have the highest number of species (Misirlioğlu et al. 2023), with approximately 476 and 135 species and subspecies in China, respectively (Zhao et al. 2025).

Megascolecid earthworms, which are characterized by having a clitellum beginning at or before XV, a single pair of male pores located on XVIII (or XVII, rarely on XIX) (Xu and Xiao 2011), are the dominant group in earthworm fauna in China. *Amynthas* and *Metaphire* species together account for 89% of total earthworm species in China (Zhao et al. 2025). However, the distribution of Megascolecidae in China is not uniform; provinces in southern China such as Sichuan, Guangxi, Taiwan, and Hainan each have records of more than 79 species (Sun 2013; Zhao 2015; Jiang 2016; Sun et al. 2018; Dong et al. 2024; Jin et al. 2024). In surveys of Megascolecidae in northern China, only 19 species/subspecies have been documented (Xu and Xiao 2011; Dong et al. 2024; Han et al. 2024, 2026; Zhao et al. 2025).

By comparison, taxonomic surveys of Megascolecidae in North China Region (including Beijing, Tianjin, Hebei, Shanxi and Shandong provinces) remain relatively limited, primarily concentrated in Beijing and Tianjin, where just 10 species/subspecies were documented, most of which are widespread, including *A. hupeiensis* (Michaelson, 1895), *A. pingi* (Stephenson, 1925), *A. carnosus carnosus* (Goto & Hatai, 1899), *A. carnosus naribunji* (Blakemore, 2013), *M. guillelmi* (Michaelsen, 1895), *M. tschiliensis* (Michaelsen, 1928), *M. vulgaris* (Chen, 1930), *M. asiatica* (Michaelsen, 1900), *M. schmardae* (Horst, 1883) and *M. houlleti* (Perrier, 1872) (Huang et al. 2006; Xu and Xiao 2011; Zhang et al. 2017; Han et al. 2024). These records primarily date from the 19^th^ and 20^th^ centuries except *A. carnosus naribunji*, which is a newly recorded species discovered in China previously (Han et al. 2024). This indicates that megascolecid diversity in China exhibits a distinct pattern of greater abundance in the south and lesser abundance in the north. Therefore, shifting the research focus to the North China region is particularly important.

Within the genus *Metaphire*, the *M. houlleti* group is one of the well-defined core groups with the following diagnostic features: three pairs of spermathecal pores in 6/7–8/9, bithecate, and post-clitellar genital markings absent (Sims and Easton 1972). The species group comprises 56 described species of which most have a wide distribution. Sims and Easton (1972) contributed the naming and descriptions of 40 species, laying a crucial foundation for the taxonomic research of this group. Additionally, ten species of this group were described in India [*M. manipurensis* Tiwari & Yadav, 2024] (Tiwari et al. 2025), Thailand [*M. khaoluangensis* Bantaowong & Panha, 2016, *M. khaochamao* Bantaowong & Panha, 2016 and *M. songkhramensis* Chanabun & Panha, 2023] (Bantaowong et al. 2016; Ratmanee et al. 2023) and Vietnam [*M. acampanulata* Nguyen, Ly, Lam, Nguyen & Nguyen, 2022, *M. amplectens* (Michaelsen, 1934), *M. honbaensis* (Gates, 1941), *M. dorsobitheca* (Thai & Huynh, 1992), *M. catbaensis* (Thai & Le, 1993) and *M. dalatensis* Phan, Lam & Nguyen, 2026] (Michaelsen 1934; Thai et al. 1992; Thai and Le 1993; Nguyen et al. 2022; Phan et al. 2026) after 1972 in southern Asia. Researches on this group in China has advanced rapidly in recent two decades, confirming 26 species, including 20 species (Michaelsen 1928; Sims and Easton 1972; Chen et al. 1975; Feng 1984; Zhong et al. 1984; Tan and Zhong 1986, 1987; Feng and Ma 1987; Qiu and Zhong 1993; Shen et al. 2005; Xu and Xiao 2011), most of which were first included in Sims and Easton (1972), and *M. sanmingensis* Sun & Jiang, 2018 (Sun et al. 2018), *M. hanbaiduensis* Dong & Sun, 2024 (Dong et al. 2024), *M. donganensis* Jin & Jiang, 2024 (Jin et al. 2024), *M. liaoningensis* Han & Zhao, 2025 (Zhao et al. 2025), *M. jinhensis* Ren, 2025 and *M. ebianensis* Ren, 2025 (Ren et al. 2025).

Earthworm classification can be challenging due to the limited availability of reliable taxonomic characters and restrictions related to the maturity of specimens (Pop et al. 2003). Since the 1990s, taxonomists have employed DNA sequences as a tool for species identification. However, the study of earthworms in this area started very late, and the first paper had not been published until 2003 (Pop et al. 2003). Nonetheless, the use of molecular data has proven to be an indispensable method for delimiting earthworm species (Pop et al. 2003; Chang and Chen 2005; Chang et al. 2007, 2008, 2009; Chang and James 2011). Among these, the mitochondrial cytochrome c oxidase subunit I (COX1) gene has been demonstrated to be highly effective and accurate for earthworm species identification (Pérez-Losada et al. 2005, 2012; Chang et al. 2009; Rougerie et al. 2009; Chang and James 2011; Porco et al. 2012).

Although COX1 barcoding served as a useful tool for species delimitation, it has been found to be limited for resolving phylogenetic relationships at above species level (Chang and James 2011). At present, the mitochondrial genome (mitogenome) has been proven to be a powerful tool in resolving earthworm phylogenetic relationships across numerous studies (Sun 2013; Zhao 2015; Zhang et al. 2016c; Sun et al. 2018; Zhao et al. 2022). Mitogenomes offer significant advantages over single-gene markers for phylogenetic studies. Their high mutation rate, absence of introns, and substantial length (> 10,000 base pairs) make them a superior tool for this purpose (Zhang et al. 2016c). The comprehensive data from mitogenomes also facilitate evolutionary studies of key genomic characteristics, such as gene content and arrangement, thereby establishing them as an essential resource for detailed and accurate earthworm phylogenetic research.

This study describes a new megascolecid species, *M. wenanensis* sp. nov., from Wen’an County, Langfang Prefecture, Hebei Province in northern China region. In addition, a phylogenetic analysis of *M. wenanensis* sp. nov. was conducted using 13 protein-coding genes (PCGs) from the mitogenomic data, aiming not only to validate its taxonomic placement but also to explore its evolutionary position within the *M. houlleti* group and to provide biogeographic context for its occurrence in northern China.

## Materials and methods

### Sampling

Earthworm specimens were collected on 10^th^ May 2023 in Xinglonggong Town, Wen’an County, Langfang Prefecture, Hebei Province, China. Earthworms were collected through digging and hand sorting. The collected earthworms were immediately preserved in 100% ethanol in the field, and then subsequently stored at −20 °C in the laboratory for subsequent morphological and molecular analyses. Both type specimen and other paratype specimens are deposited in Langfang Normal University, Hebei, China (**C-HLU**).

### Morphological examination

External and internal morphological characters of 14 clitellate specimens (voucher ID: LFWA_01–14) were examined using a stereomicroscope (ZEISS) and ZEN 3.3. pro software for image capturing and to aid in identifying and measuring small organs and other characters for morphological analysis. Body length and diameter were measured; and morphological characters such as the male and female pores, the spermathecal pore, spermathecae, prostate gland, gizzard and caeca were examined and recorded (measurements in mm). The species diagnoses and taxonomic assignments follow (Sims and Easton 1972; Xu and Xiao 2011).

### DNA extraction, amplification, and sequencing

The total genomic DNA was extracted from the posterior region of each of the 21 specimens utilizing the TIANGEN Genomic DNA Kit DP304-03 (Beijing, China). Subsequently, high-throughput sequencing was performed on the specimen designated as LFWA_01 of *M. wenanensis* sp. nov. and seven individuals of both *M. sanmingensis* and *M. tschiliensis*, employing a paired-end 150 bp sequencing strategy on the DNBseq platform at BGI Genomics (Wuhan, China). The subsequent filtration of clean data from the raw sequencing data was conducted in accordance with the procedures delineated by (Zhao et al. 2022). The mitogenomic sequence was assembled and mapped using MitoZ v2.4 (Meng et al. 2019) and annotated with the online tool MITOS2 (https://usegalaxy.org/root?tool_id=toolshed.g2.bx.psu.edu%2Frepos%2Fiuc%2Fmitos2%2Fmitos2%2F2.1.3%20galaxy0). The mitogenomic sequence and its annotation were subsequently submitted to GenBank (accession numbers are provided in Table 1).

**Table 1.** Specimens’ information provided and available online DNA data analyzed in this study.

Specimen ID	Species	Genetic marker	Accession no.	References
LFWA_02– LFWA_14	*M. wenanensis* sp. nov.	COX1	PX552892–PX552903	This study
-	*M. tschiliensis*	COX1	PP527638	Zhao et al. 2025
-	*M. tschiliensis*	COX1	PP527667	Zhao et al. 2025
-	*M. tschiliensis*	COX1	PP527575	Zhao et al. 2025
-	*M. liaoningensis*	COX1	PP527733– PP527735	Zhao et al. 2025
-	*M. guillelmi*	COX1	PP527738	Zhao et al. 2025
-	*M. guillelmi*	COX1	KT429017	Zhang et al. 2016c
-	*M. vulgaris*	COX1	KJ137279	Zhang et al. 2016a
-	*M. vulgaris*	COX1	PQ720812	Bi et al. 2025
-	*M. trutina*	COX1	AY960808	Chang et al. 2008
-	*M. trutina*	COX1	AY962148	Chang et al. 2008
-	*M. sanmingensis*	COX1	KY774382– KY774384	Sun et al. 2018
-	*M. donganensis*	COX1	PP497092– PP497094	Jin et al. 2024
LFWA_01	*M. wenanensis* sp. nov.	mitogenome	PX369608	This study
L319FLT03	*M. sanmingensis*	mitogenome	PZ099867	This study
533R55_01	*M. tschiliensis*	mitogenome	PP507116	This study
XLM_03	*M. tschiliensis*	mitogenome	PP504842	This study
LFSF009	*M. tschiliensis*	mitogenome	PP504841	This study
534R1_08	*M. tschiliensis*	mitogenome	PP507117	This study
HNLN_GR_I1_10	*M. tschiliensis*	mitogenome	PP504838	This study
HNLN_GR_I3_17	*M. tschiliensis*	mitogenome	PP504840	This study
-	*M. tschiliensis*	mitogenome	PP504839	Zhao et al. 2025
-	*M. liaoningensis*	mitogenome	PP507115	Zhao et al. 2025
-	*M. liaoningensis*	mitogenome	PP504837	Zhao et al. 2025
-	*A. gracilis*	mitogenome	KP688582	Zhang et al. 2015
-	*A. corticis*	mitogenome	KM199290	Zhang et al. 2015
-	*A. aspergillum*	mitogenome	KJ830749	Zhang et al. 2016a
-	*M. vulgaris*	mitogenome	KJ137279	Zhang et al. 2016a
-	*A. aspergillum*	mitogenome	OR161103	Zhang et al. 2016b
-	*A. triastriatus*	mitogenome	KT429016	Zhang et al. 2016c
-	*M. guillelmi*	mitogenome	KT429017	Zhang et al. 2016c
-	*A. carnosus*	mitogenome	KT429008	Zhang et al. 2016c
-	*M. posthuma*	mitogenome	MW222472	Yu et al. 2022
-	*A. deogyusanensis*	mitogenome	OK558822	Koo and Hong 2023
-	*M. megascolidioides*	mitogenome	LC726529	Sato et al. 2023
-	*M. tosaensis*	mitogenome	LC726512	Sato et al. 2023
-	*A. yambaruensis*	mitogenome	LC726555	Sato et al. 2023
-	*A. carnosus*	mitogenome	LC726524	Sato et al. 2023
-	*A. robustus*	mitogenome	LC726556	Sato et al. 2023
-	*M. californica*	mitogenome	LC726499	Sato et al. 2023
-	*A. hupeiensis*	mitogenome	LC726513	Sato et al. 2023
-	*A. tappensis*	mitogenome	LC726521	Sato et al. 2023
-	*A. tokioensis*	mitogenome	LC726526	Sato et al. 2023
-	*M. agrestis*	mitogenome	LC726525	Sato et al. 2023
-	*A. glaucus*	mitogenome	LC726553	Sato et al. 2023
-	*Polypheretima elongata*	mitogenome	LC726551	Sato et al. 2023
-	*Perionyx excavatus*	mitogenome	EF494507	Kim et al. 2005

COX1 was amplified via polymerase chain reaction (PCR), utilizing the forward primer of LCO1490 (5’-GGTCAACAAATCATAAAGATATTGG-3’) (Folmer et al. 1994) and reverse primer COIE (5’-TATACTTCTGGGTGTCCGAAGAATCA-3’) (Bely and Wray 2004). The PCR mixture, with a total volume of 25 μl, consisted of 1 μl of DNA template, 17.25 μl of sterile distilled water, 2.0 μl of dNTP mixture, 2.5 μl of reaction buffer, 0.25 μl of Easy Taq Polymerase (TransGen Biotech Co., LTD, Beijing, China), 1.0 μl of LCO1490, and 1.0 μl of COIE. The thermal cycling parameters were configured as follows: an initial denaturation step at 95 °C for 5 minutes, followed by 35 cycles consisting of denaturation at 95 °C for 30 seconds, annealing at 51 °C for 30 seconds, and extension at 72 °C for 30 seconds. The process concluded with a final extension step at 72 °C for 5 minutes. Amplified PCR products were sent to Tianyi Huiyuan Biotechnology Co., Ltd. (Beijing, China) for Sanger sequencing. COX1 sequences were deposited in GenBank, and additional sequences included in this study were retrieved from GenBank (accession numbers see Table 1). The genetic distances among these sequences were calculated using the Kimura 2-parameter (K2P) model (Kimura 1980), which was implemented in the MEGA 5.0 (Tamura et al. 2011).

### Molecular species delimitation analyses

To preliminarily infer species hypotheses, two methods were applied to the COX1 marker: the distance matrix method of Assemble Species by Automatic Partitioning (ASAP) (Puillandre et al. 2021), and the phylogenetic method of Generalized Mixed Yule Coalescent (GMYC) (Fujisawa and Barraclough 2013).

ASAP is an automated species delimitation method based on COX1 and is designed to identify unique barcode gaps, that is, thresholds of genetic distance, thereby determining whether two individuals belong to the same species. One of the ASAP main qualities is that it is extremely fast compared to any method that relies on tree reconstruction, and is performed online (https://bioinfo.mnhn.fr/abi/public/asap). ASAP is also less prone to mismatches.

GMYC is a statistical approach for species delimitation using single-locus data. It distinguishes species by analyzing branching patterns in gene trees, applying a Yule model for diversification between species and a neutral coalescent model (Hudson 1991) for branching within species. The GMYC analysis was performed using the package splits v. 1.0-19 (Ezard et al. 2017) in R. The input files for GMYC were prepared using the BEAST package v. 1.7.5 (Drummond et al. 2012), including setting a Yule Process tree prior and a lognormal relaxed clock model. Newick-formatted ultrametric, bifurcating, and rooted trees were generated with TreeAnnotator (included in the BEAST package) and provided as input for the GMYC analysis. The final tree was visualized in FigTree v. 1.4.4 (Rambaut 2022) (http://tree.bio.ed.ac.uk/software/figtree/)

### Phylogenetic analysis

Mitogenomic data from 34 earthworm taxa were used to infer the phylogenetic position of *M. wenanensis* sp. nov. among closely related taxa (accession numbers in Table 1). *Perionyx excavatus* Perrier, 1872 served as the outgroup. Thirteen PCGs were extracted from the GenBank file using the script gbseqextractor_v2.py (Meng et al. 2019). Phylogenetic reconstruction was conducted using both maximum likelihood (ML) and Bayesian inference (BI) frameworks. ML analysis was implemented in RAxML 8.0 (Stamatakis 2014) employing the rapid hill-climbing algorithm under the GTRGAMMAI model to identify optimal tree topologies, with clade support evaluated through 1,000 rapid bootstrap replicates. For Bayesian analysis with MrBayes v. 3.2.6 (Ronquist et al. 2012) two million Markov chain Monte Carlo (MCMC) generations were executed, continuing until the average standard deviation of split frequencies fell below 0.01. Nucleotide substitution models were selected via jModelTest 2.1 (Darriba et al. 2012) using the Akaike Information Criterion, resulting in GTR+I+G for PCGs. Convergence diagnostics and burn-in assessment were performed in Tracer v. 1.7.2 (Rambaut et al. 2018), where all parameters exhibited effective sample sizes (ESS) exceeding 200.

## Results

### Taxonomy

**Family Megascolecidae Rosa, 1891**

**Genus *Metaphire* Sims & Easton, 1972**

#### Metaphire wenanensis

Taxon classificationAnimaliaCrassiclitellataMegascolecidae

Liu & Zhao sp. nov.

AAAA7A8E-2D29-512B-81B1-E42DCB2B0C4A

https://zoobank.org/D80A893A-443F-4D7D-B21E-B3AA7F4F488D

Figs 1, 2, 3, 4

##### Material examined.

***Holotype***: • One clitellate (LFWA_01), collected in Xinglonggong Town (38.90973°N, 116.23225°E, 5 m elevation.), Wen’an County, Langfang Prefecture, Hebei Province. Collection date: 2023-05-10. Collector: Huifeng Zhao. ***Paratypes***: • Five clitellates [LFWA_02, 03, 05–07], same data as of holotype.

##### Additional specimens.

• Eight clitellates (LFWA_04, 08–14), same data as of holotype. All types and other specimens are preserved in C-HLU.

##### Diagnosis.

Medium sized, length 90–105 mm, diameter 7–10 mm, number of segments 105–110. Prostomium epilobous. Dorsal pores between segments 12/13. Three pairs of spermathecal pores located ventrally between segments 6/7–8/9. Male pores on the ventral sides of XVIII, spaced ~ 1/3 around the body. Female pore singular, on the ventral side of segment XIV. Clitellum annular, spanning segments XIV–XVI, and lacks setae. Setae perichaetine arrangement. Genital markings absent. Gizzard in IX–X. Caeca beginning at XXVII, extending forward to XXIII. Three pairs of spermathecae in VII–IX; ampulla nearly elliptical, smooth-surfaced, ampulla duct long, stout with swollen basal portion. Diverticulum originating from below the swollen portion of the spermathecal duct, stalk slender at the proximal end, enlarged and greatly coiled toward distal end. Seminal vesicles in XI–XII. Pair of prostate glands well-developed, in segments XVI–XIX, follicular, with a thin U-shaped prostatic duct.

##### Description.

**Figure 1.**
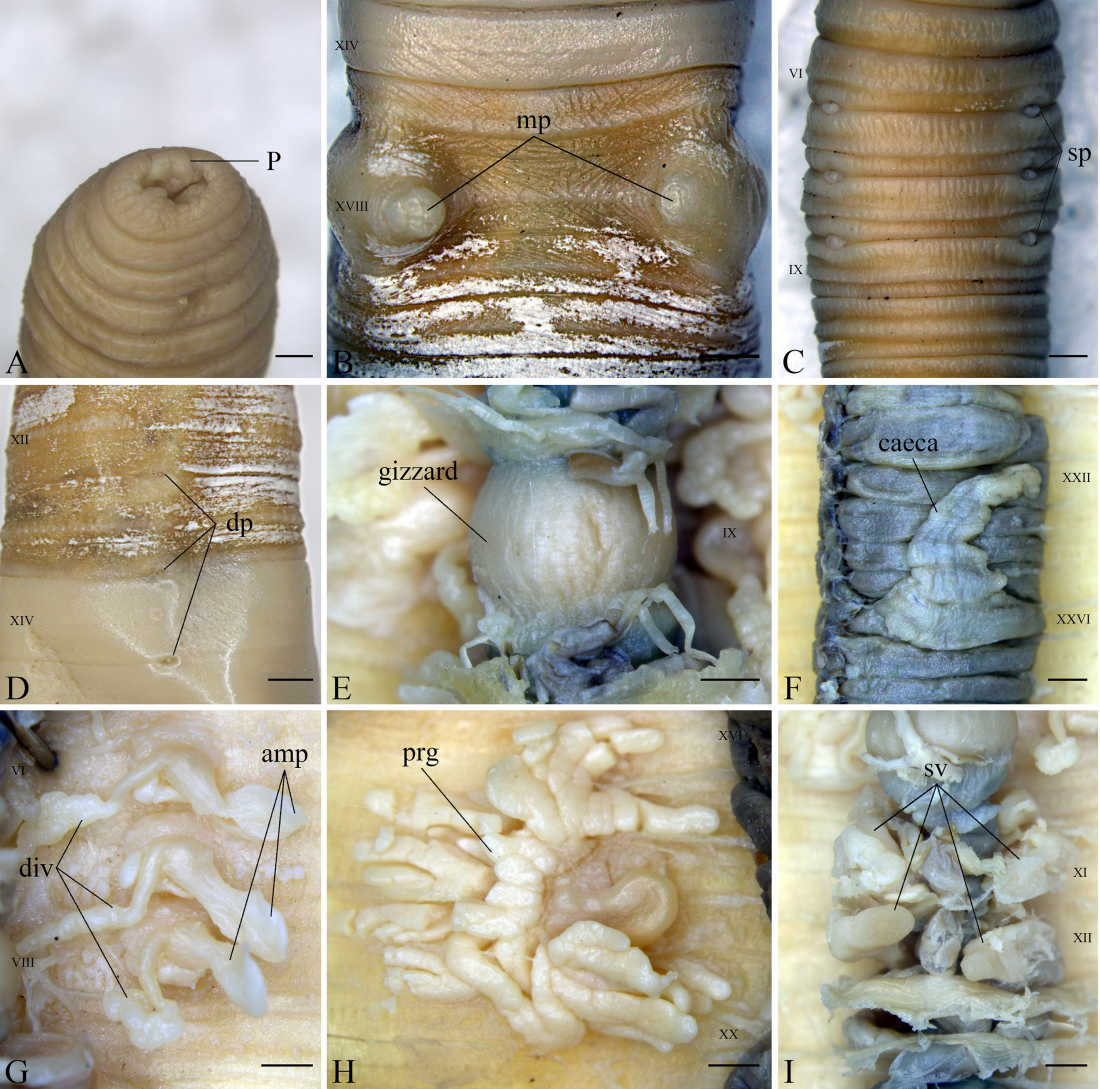
*Metaphire wenanensis* sp. nov., holotype (LFWA_01). **A**. Prostomium; **B**. Ventral view of the male pores; **C**. Ventral view of the spermathecal region; **D**. Dorsal pores; **E**. Gizzard; **F**. Ventral intestinal caeca; **G**. Right spermathecae; **H**. Left prostate gland; **I**. Seminal vesicles. Abbreviations: amp = Ampulla; div = Diverticulum; dp = Dorsal pore; mp = Male pore; p = Prostomium; prg = Prostate gland; sp = Spermathcal pore; sv = Seminal vesicles. Scale bars: 1 mm.

***External characters***. Length 90–105 mm. Body color yellowish-white, dorsal side darker than the ventral side; clitellum pale yellowish-white color. Width 7–10 mm, segments 105–110. Prostomium epilobous (Fig. 1A). First dorsal pore 12/13 (Fig. 1D). Clitellum annular XIV–XVI; setae absent. Setal arrangement perichaetine; setae number 42 (V), 48 (XII), 56(XX). Setae between male porophores: 10–11. Female pore single in slit-like opening, medioventral at XIV.

**Figure 2.**
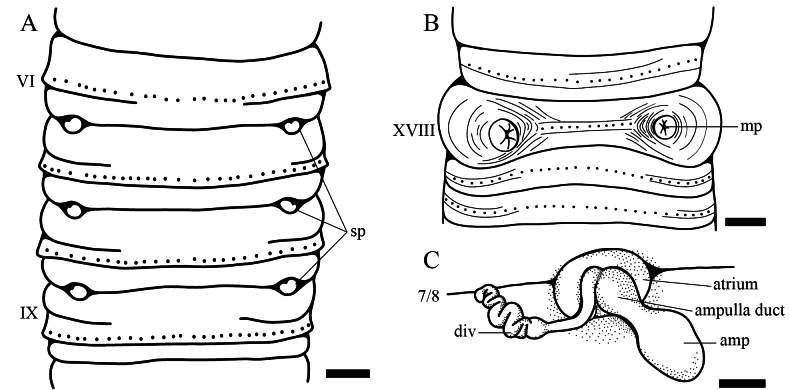
Line drawing of *Metaphire wenanensis* sp. nov., holotype (LFWA_01). **A**. Ventral view of the spermathecal region; **B**. Male pores; **C**. A right spermathecae. Abbreviations: amp = Ampulla; div = Diverticulum; mp = Male pore; sp = Spermathecal pore. Scale bars: 1 mm.

Spermathecal pores three pairs, 6/7–8/9, ventrolateral; Small, round, and slightly sunken into the body surface. Ventral distance between openings of the spermathecal pores ~ 3.4–4.2 mm (Fig. 1C). Pores bearing spherical projections. Pre-clitellar genital markings absent (Fig. 2A).

Male pores paired on segment XVIII (Fig. 1B), each located within a copulatory pouch. The copulatory pouches are slightly raised on the body wall, surrounded by two or three circles of cutaneous folds. Internally, each copulatory pouch encloses the male pore and the opening of the prostatic duct. The ventral distance between the two male pore openings is 3.7–4.5 mm. Post-clitellar genital marking absent (Fig. 2B).

***Internal characters***. Septa 8/9/10 aborted, 4/5/6/7/8 thick and muscular, 10/11/12/13 uniform thickness. Gizzard within IX–X, small (Fig. 1E). Intestine enlarged from XIV. Intestinal caeca simple, originating in XXVII and extending anteriorly to XXIII; however, some caeca extend up to XXII (Fig. 1F).

Spermathecae three pairs in VII–IX (Fig. 1G), ampulla nearly elliptical, small, surface flattened and smoothed, 1.09–2.56 mm long, 1.37–1.93 mm wide; ampulla duct long, stout, 0.91–1.52 mm long, with swollen basal portion, 0.87–1.04 mm wide; diverticulum originating from disc-shaped atrium connected below swollen portion of ampulla duct, stalk slender at the proximal end 1.41–2.50 mm long, enlarged and greatly coiled toward distal end, receptacle is 1.09–3.01 mm long. Diverticulum connected to the ampulla duct at a disc-shaped atrium. The atrium area 1.66–2.48 mm^2^, attaching to body wall (Fig. 2C).

Testis sac one pair in XI. Seminal vesicles two pairs in XI and XII, well developed, follicular, posterior pair larger (Fig. 1I). Prostate glands large, paired in XVIII (Fig. 1H), follicular, divided into many finger like projections, extending anteriorly to XVI (or XVII) and posteriorly to XX (or XXI). Prostatic duct U-shaped, slender.

##### Etymology.

The species name refers to the type locality in Wen’an County, Langfang Prefecture, Hebei Province.

##### Distribution.

Northern China (Hebei)

##### Habitats.

Specimens were collected from riparian soil along the banks of a freshwater river. The soil type is sandy loam rich in organic matter, collected at a depth of ~ 20 cm. The vegetation cover is dominated by willow trees (*Salix* spp.).

##### Remarks.

*Metaphire wenanensis* sp. nov. belongs to the *M. houlleti* group, which has ~ 56 species. This group is characterized by having three pairs of bi-chambered spermathecal pores at segments 6/7–8/9, with consistent patterns in pore placement and clitellum morphology. The new species is morphologically similar to *M. tschiliensis*; *M. vulgaris* and *M. guillelmi*. Key distinguishing features of *M. wenanensis* sp. nov. include: first dorsal pores starting at segment 12/13 (versus 11/12 in *M. vulgaris* and *M. tschiliensis*), absence of pre-clitellar and post-clitellar genital markings, inconspicuous female pores (contrasting with the single ventral pore on XIV in *M. tschiliensis*) (Table 2).

**Table 2.** Comparison of species belonging to the *M. wenanensis* sp. nov. Hyphen indicates not available.

Character	*M. wenanensis* sp. nov.	*M. sanmingensis*	*M. donganensis*	*M. tschiliensis*	*M. vulgaris*	*M. liaoningensis*	*M. guillelmi*
Length (mm)	90–105	55–113	72–159	155–240	120–215	125–190	96–150
Width (mm)	7–10	4–5.5	4.6–7	6.5–7	5–8	7	5–8
No. of segments	105–110	55–86	78–111	111–165	90–124	120–145	88–156
First dorsal pore	12/13	11/12, or 12/13, or 13/14	12/13	11/12 or 12/13	11/12	12/13	12/13 or 13/14
Setae on V, XIII, XX	42(V)	23–32(V)	-(V)	45–50 (V)	-	43 (V)	-(V)
	48(XIII)	30–40(VIII)	28–58(VIII)	70–72 (XIII)		70 (XIII)	-(VIII)
	56(XX)	44–48(XX)	52–66(XX)	-(XX)		-(XX)	44–64(XX)
Spermathecae pores, distance apart	0.21–0.27C	0.33C	0.33C	0.29–0.30 C	-	0.28–0.30 C	-
Male pore, distance apart	0.24–0.29C	0.33C	0.33C	2/5 circumference apart	-	-	-
Setae between male pore	10–11	8–9	18–13	10–17	12–22	12–20	14–21
Pre-clitellar genital marking	absent	present	absent	present or absent	present	present	absent
Post-clitellar genital marking	absent	present	absent	absent	present	absent	absent
Gizzard	IX–X	X–XI	IX–X	IX–X	IX–X	IX–X	VIII–IX
Caeca	XXVII–XXIII (or some XXII)	XXVII–XXIV	XXVII–XXIII	XXVII–XXIII	XXVII–XXIII	XXVII–XXIII (or some XX)	XXVII to XXII
Accessory glands	absent	absent	absent	absent	present	present	absent
Atrium base of spermathecae	present	absent	absent	absent	absent	absent	absent

*Metaphire wenanensis* sp. nov. is also morphologically similar to *M. liaoningensis*. Both species have the first dorsal pores commencing at segment 12/13 and have paired prostates at segment XVIII in a follicular form. Similarities extend to the number and placement of male pores, spermathecae, and gizzards. However, the two species differ in the genital markings and accessory glands: in *M. wenanensis* sp. nov. the pre-clitellar genital markings and accessory glands are absent, while in *M. liaoningensis*, the pre-clitellar genital markings are present, with three pairs of papillae presetal on VII, XIII, and IX, and three pairs of accessory glands in VII–IX.

The spermathecal features of the new species are highly distinctive. Unlike all other members of the *M. houlleti* group, where the ampulla duct and diverticulum connect directly to the body wall, *M. wenanensis* sp. nov. possesses a disc-shaped atrium interposed between these structures and the body wall. This unique anatomical feature is potentially associated with the spherical swelling observed at each spermathecal pore. To the best of our knowledge, this feature is unique among all compared species of the *M. houlleti* group.

### Molecular species delimitation

The K2P analysis based on the COX1 gene reveals an intraspecific genetic distance of *M. wenanensis* sp. nov. ranging from 0 to 2.1%. The interspecific genetic distance between the new species and the other species in the *M. houlleti* species group ranges from 15.2% (compared to *M. donganensis*) to 19.9% (compared to *M. vulgaris*) (Table 3).

**Table 3.** K2P distances of COX1 of *M. wenanensis* sp. nov. with other members of the *M. houlleti* species group. Bold numbers show an intra-species genetic distances.

Species		1	2	3	4	5	6	7	8
1	*M. donganensis*	**0.2**–**5.4%**							
2	*M. wenanensis* sp. nov.	15.2–17.2%	**0**–**2.1**%						
3	*M. guillelmi*	18.8–19.1%	18.8–19.1%	**0**					
4	*M. liaoningensis*	17.6–18.1%	19.2–19.4%	13.6–13.9%	**0–0.2%**				
5	*M. sanmingensis*	18.7–19.7%	18.5–18.7%	18.3%	18.8–19.1%	**0**			
6	*M. trutina*	17.0–22.4%	18.6–19.8%	18.0–18.3%	18.3–18.7%	17.5–18.7%	**0**–**7.5%**		
7	*M. tschiliensis*	17.0–19.1%	17.0–18.5%	16.7–18.1%	15.7–16.7%	17.7–18.7%	18.0–20.1%	**5.1**–**7.5%**	
8	*M. vulgaris*	17.6–18.6%	19.6–19.9%	7.1%	15.9–16.1%	19.4%	18.1–19.1%	15.9–17.6%	**0**

**Figure 3.**
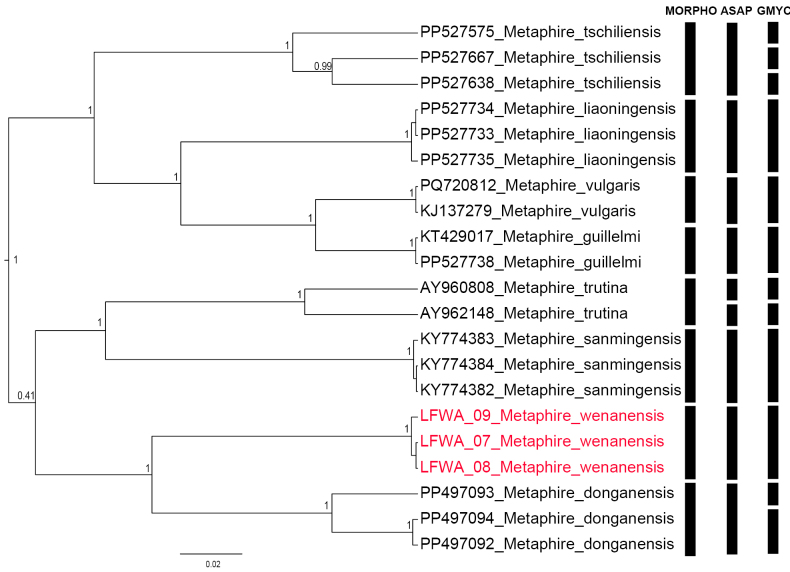
Species delimitation of the *M. houlleti* group based on COX1 sequences. MORPHO, morphological delimitation; ASAP, automated barcode gap discovery; GMYC, generalized mixed Yule coalescent model. Numbers near branches indicate the Bayesian posterior probabilities. The new species is indicated in red.

ASAP employing the COX1 gene delineates species into nine Molecular Operational Taxonomic Units (MOTUs), which is one MOTU more than the morphological classification (Fig. 3). The difference lies in that ASAP assigned the morphologically classified *M. trutina* into two MOTUs. In comparison, GMYC delineates species into twelve MOTUs. Specifically, it assigned the morphologically classified *M. tschiliensis* into three MOTUs, while *M. trutina* and *M. donganensis* are each assigned into two MOTUs. Although ASAP shows minor discrepancies compared to morphological classification, it demonstrates better performance than GMYC, a finding corroborated by other molecular species delimitation studies on earthworms (Goulpeau et al. 2022; Liu et al. 2025a, 2025b). While there are slight differences between the results of ASAP, GMYC, and morphological classification, all three methods display high consistency in the delimitation of *M. wenanensis* sp. nov. designating it as a single MOTU distinct from other species. Therefore, *M. wenanensis* sp. nov. is recognized as a distinct new species within the *M. houlleti* group, separating it from the other members.

### Mitogenome characterization

**Figure 4.**
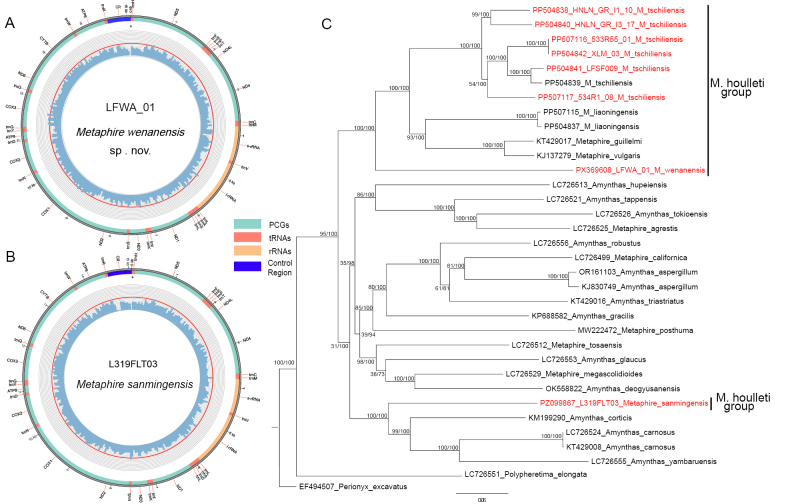
**A, B**. Mitogenomes of *M. wenanensis* sp. nov. and *M. sanmingensis*; **C**. Phylogenetic relationship of *M. wenanensis* sp. nov. and other species in the *M. houlleti* species group using the 13 coding genes of mitogenome. Numbers near branches indicate the maximum likelihood bootstrap support/Bayesian posterior probabilities. The new species is indicated in red.

The complete mitochondrial genome of *M. wenanensis* sp. nov. (holotype, LFWA_01) is a circular molecule of 15,092 bp in length (Fig. 4A). It contains 37 genes, including 13 protein-coding genes (PCGs, 11,117 bp), two ribosomal RNA genes (rRNAs, 2,047 bp), 22 transfer RNA genes (tRNAs, 1,400 bp), and one putative control region (528 bp). The gene arrangement of the mitogenome is identical to that of other members of the *M. houlleti* group (Fig. 4A, B) (Zhang et al. 2016a; Zhao et al. 2025).

### Phylogenetic analysis

The mitogenomes of *M. wenanensis* sp. nov. and *M. sanmingensis* are visualized (Fig. 4A, 4B). Combined with the publicly available data, the 13 mitogenomic PCGs revealed the phylogenetic relationship between *M. wenanensis* sp. nov. and the other species in the *M. houlleti* group (Fig. 4C). The resulting tree shows that *M. tschiliensis*, *M. vulgaris*, *M. guillelmi*, *M. liaoningensis*, and *M. wenanensis* sp. nov. form a strongly supported monophyletic clade (posterior probability = 100%, bootstrap value = 100%), while *M. sanmingensis* is clustered outside this clade. This finding suggests that the *M. houlleti* group, as presently defined, may not be monophyletic taxa. Within the main clade, *M. wenanensis* sp. nov. represents a distinct lineage clearly separated from the other four species, supporting its status as a new species. Meanwhile, the new species is relatively a basal lineage in the main *M. houlleti* group, although not all species are included as the limited mitogenome resources in the group as more than 50 species in houlleti group have no mitogenomic data.

## Discussion

This study describes *M. wenanensis* sp. nov., the first species of Megascolecidae reported from northern China, particularly in Wen’an County, Hebei Province in almost a century.

Molecular data provided robust support for species delimitation. The K2P distances between *M. wenanensis* sp. nov. and other members of the *M. houlleti* group (15.2–19.9%) substantially exceed the 13% threshold proposed by Jeratthitikul et al. (2017) as the minimum genetic distance for species differentiation in earthworms and are also higher than the values reported by Nguyen et al. (2021) for interspecific divergence within the genus *Metaphire*. This further supports the recognition of *M. wenanensis* sp. nov. as a distinct species. Both ASAP and GMYC methodologies delimit the new species as an independent MOTU. Meanwhile, morphological characters also support the species status of the new species belonging to the *M. houlleti* group. This species group is distinguished by a unique disc-shaped atrium at the spermathecal base among congeners. (Xu and Xiao 2011; Jiang 2016; Han 2025)

It should be noted that the generic distinction between *Metaphire* and *Amynthas* based on the presence or absence of copulatory pouches has been challenged by molecular phylogenetic studies, which consistently recover these genera as non-monophyletic (Zhang et al. 2015). Despite this, morphology remains the primary basis for species description and identification in earthworm taxonomy, and no comprehensive revision of the group has yet been proposed. In the new species described here, copulatory pouches are well developed and clearly visible in dissections, satisfying the traditional morphological criterion for placement in *Metaphire* (Sims and Easton 1972). Therefore, following current taxonomic convention, we assign *M. wenanensis* sp. nov. to *Metaphire*. We acknowledge that future integrative studies incorporating broader molecular and morphological data may eventually lead to a revised classification of these genera.

Phylogenetic analysis based on the limited mitogenomic data currently available (five of 56 described species) suggests that *M. wenanensis* sp. nov. may represent a basal lineage within the *M. houlleti* group (Fig. 4). However, the available data also indicates that *M. sanmingensis* does not cluster with *M. wenanensis* sp. nov., *M. tschiliensis*, *M. guillelmi*, and *M. vulgaris* (Fig. 4C), suggesting that the *M. houlleti* group does not form a monophyletic assemblage as currently perceived. This observation further underscores the provisional nature of the present phylogenetic framework. Due to the limited taxon sampling, these placements and relationships should be considered preliminary. The geological and biogeographical observations discussed below are offered as contextual background that is consistent with, but does not independently demonstrate, a potential ancient origin for this species.

First, the type locality is situated within the Haihe River system, a key hydrological framework during the formation and evolution of the North China Plain. With its fluvial pattern initially taking shape in the Neogene, this river system boasts a relatively long geological history (Wu et al. 2008), potentially providing a stable long-term habitat for ancient soil faunal assemblages.

Second, Megascolecidae constitutes an evolutionarily ancient lineage that completed its primary radiation by the Early Jurassic (Qiu and Bouche 1998). Species-level diversification subsequently commenced during the Late Cretaceous, with ancestral lineages colonizing China from the Late Cretaceous to early Cenozoic (Jiang and Qiu 2018). The occurrence of a potential relict clade of this family within the North China Plain, a geologically stable craton of Precambrian origin, is therefore phylogenetically and biogeographically congruent. Future studies incorporating broader mitogenomic sampling across a wider representation of the *M. houlleti* group are needed to robustly test evolutionary relationships and revise the group’s definition, given that preliminary data indicates its non-monophyly.

## Conclusions

This study reports a novel species, *M. wenanensis* sp. nov., within the *M. houlleti* group and provides data on its COX1 gene and mitochondrial genome. The new species is distributed in Wen’an County, Hebei Province, representing the first record of a new species of the family Megascolecidae in northern China. Megascolecid earthworms in China exhibit an uneven distribution, with more records from Southern China. Future research should focus on expanding surveys in northern China and across the country to better understand the geographic distribution of *M. wenanensis* sp. nov. and to enhance the taxonomic and distributional knowledge of megascolecid earthworms in the northern Chinese region.

## Supplementary Material

XML Treatment for Metaphire wenanensis

## Additional information

### Artificial Intelligence (AI) use

The authors accept full responsibility for the content of the manuscript, including the disclosure of any use of AI.

No AI tools were used in the preparation of this manuscript.

### Author ORCIDs

Q. Liu https://orcid.org/0009-0009-1325-2251

H. Zhao https://orcid.org/0000-0003-4243-9671

N.M. Aspe https://orcid.org/0000-0003-0025-6017

Y. Zhang https://orcid.org/0000-0002-4066-6355

Y. Dong https://orcid.org/0009-0004-3360-3673

### Data availability

All of the data that support the findings of this study are available in the main text.

## References

[B1] Bantaowong U, Chanabun R, James SW, Panha S (2016) Seven new species of the earthworm genus *Metaphire* Sims & Easton, 1972 from Thailand (Clitellata: Megascolecidae). Zootaxa 4117: 63–84. 10.11646/zootaxa.4117.1.3 27395158

[B2] Bely AE, Wray GA (2004) Molecular phylogeny of naidid worms (Annelida: Clitellata) based on cytochrome oxidase I. Molecular Phylogenetics and Evolution 30: 50–63. 10.1016/s1055-7903(03)00180-5 15022757

[B3] Bi Y, Fan Y, Cheng X, Li N, Wang S, Zhang H, Zhang Y, Zhao H (2025) Earthworm Diversity of Tianjin, China. Chinese Journal of Soil Science 56: 1–8. 10.19336/j.cnki.trtb.2025012201

[B4] Chang C-H, Chen J-H (2005) Taxonomic status and intraspecific phylogeography of two sibling species of *Metaphire* (Oligochaeta: Megascolecidae) in Taiwan. Pedobiologia 49: 591–600. 10.1016/j.pedobi.2005.07.002

[B5] Chang C-H, James S (2011) A critique of earthworm molecular phylogenetics. Pedobiologia 54: S3–S9. 10.1016/j.pedobi.2011.07.015

[B6] Chang C-H, Lin S-M, Chen J-H (2008) Molecular systematics and phylogeography of the gigantic earthworms of the *Metaphire formosae* species group (Clitellata, Megascolecidae). Molecular Phylogenetics and Evolution 49: 958–968. 10.1016/j.ympev.2008.08.025 18809504

[B7] Chang C-H, Lin Y-H, Chen IH, Chuang S-C, Chen J-H (2007) Taxonomic re-evaluation of the Taiwanese montane earthworm *Amynthas wulinensis* Tsai, Shen & Tsai, 2001 (Oligochaeta: Megascolecidae): Polytypic species or species complex? Organisms Diversity & Evolution 7: 231–240. 10.1016/j.ode.2006.06.001

[B8] Chang C-H, Rougerie R, Chen J-H (2009) Identifying earthworms through DNA barcodes: Pitfalls and promise. Pedobiologia 52: 171–180. 10.1016/j.pedobi.2008.08.002

[B9] Chen Y, Xu Z, Yang T, Feng X (1975) On some new earthworms from China. Acta Zoologica Sinica 21: 89–99.

[B10] Darriba D, Taboada GL, Doallo R, Posada D (2012) jModelTest 2: more models, new heuristics and parallel computing. Nature Methods 9: 772. 10.1038/nmeth.2109 PMC4594756 22847109

[B11] Dong Y, Jiang J, Zhang J, Shen Z, Sun J (2024) Three New Earthworm Species of the Genus *Metaphire* (Oligochaeta: Megascolecidae) From Eastern and Southern China. Chinese Journal of Zoology 59: 397–407. 10.13859/j.cjz.202423079

[B12] Drummond A, Suchard M, Xie D, Rambaut A (2012) Bayesian phylogenetics with BEAUti and the BEAST 1.7. Molecular Biology and Evolution 29(8): 1969–1973. 10.1093/molbev/mss075 PMC3408070 22367748

[B13] Ezard TH, Fujisawa T, Barraclough TG (2017) split: Species Limits and Phylogenetic Analysis (Version 1.0-19) [R Package].

[B14] Feng X (1984) A new subspecies of terrestrial Oligochaeta from Lanzhou, Kansu Province. Zoological Research 5: 47–50.

[B15] Feng X, Ma Z (1987) Notes on a new species of the genus *Metaphire* from Gansu Province, China (Oligochaeta: Megascolecidae). Acta Zootaxonomica Sinica 12: 248–250.

[B16] Folmer O, Black M, Hoeh W, Lutz R, Vrijenhoek R (1994) DNA primers for amplification of mitochondrial cytochrome c oxidase subunit I from diverse metazoan invertebrates. Molecular Marine Biology and Biotechnology 3: 294–299 7881515

[B17] Fujisawa T, Barraclough TG (2013) Delimiting Species Using Single-Locus Data and the Generalized Mixed Yule Coalescent Approach: A Revised Method and Evaluation on Simulated Data Sets. Systematic Biology 62(5): 707–724. 10.1093/sysbio/syt033 PMC3739884 23681854

[B18] Goulpeau A, Penel B, Maggia M-E, Marchán DF, Steinke D, Hedde M, Decaëns T (2022) OTU Delimitation with Earthworm DNA Barcodes: A Comparison of Methods. Diversity 14: 866. 10.3390/d14100866

[B19] Han ACN (2025) Integrative Taxonomy and Distribution of *Pheretimoid* Earthworms in Northeastern China. Master thesis. Northeast Normal University. [In English]

[B20] Han ACN, Zhang Y, Miao P, Wu S, Xiao N, Qin M, Zhao H, Wu D, Aspe NM (2024) Distribution and systematics of the cosmopolitan *Amynthas carnosus* complex (Crassiclitellata, Megascolecidae) from eastern Asia. Zoosystematics and Evolution 100: 1061–1073. 10.3897/zse.100.119292

[B21] Han CAN, Zhao N, Aspe NM, Nob CJR, Zhang Y, Wu D, Zhao H (2026) A new species of the *Amynthas corticis* group with support from mitogenomic data and a new record of *Metaphire agrestis* (Goto & Hatai, 1899) (Oligochaeta, Megascolecidae) in northeastern China. ZooKeys 1266: 231–261. https//doi.org/10.3897/zookeys.1266.161903 10.3897/zookeys.1266.161903 PMC12817059 41568028

[B22] Huang J, Xu Q, Sun Z, Wang C, Zheng D (2006) Research on earthworm resources of China: I. Checklist and distribution. Journal of China Agricultural University: 9–20.

[B23] Hudson RR (1991) Gene genealogies and the coalescent process. In: Futuyma D, Antonovics J (Eds) Oxford Surveys in Evolutionary Biology. Oxford University Press, New York, 1–44.

[B24] Jeratthitikul E, Bantaowong U, Panha S (2017) DNA barcoding of the Thai species of terrestrial earthworms in the genera *Amynthas* and *Metaphire* (Haplotaxida: Megascolecidae). European Journal of Soil Biology 81: 39–47. 10.1016/j.ejsobi.2017.06.004

[B25] Jiang J (2016) Taxonomy and molecular phylogeny of the family Megascolecidae earthworms from China. Doctoral dissertation, Shanghai Jiao Tong University. [In Chinese with English abstract]

[B26] Jiang J, Qiu J (2018) Origin and evolution of earthworms belonging to the family Megascolecidae in China. Biodiversity Science 26: 1074–1082. 10.17520/biods.2018105

[B27] Jiang J, Qiu J (2024) The Resource and Conservation of Earthworms in China. Science China 76: 47–52.

[B28] Jin Q, Li J, Jiang J, Qiu J (2024) Four new earthworm species of the genera *Amynthas* and *Metaphire* (Oligochaeta, Megascolecidae) from Hunan and Anhui provinces, China. ZooKeys 1210: 247–271. 10.3897/zookeys.1210.125963 PMC11369497 39228391

[B29] Kim D, Lee K, Jee S, Seo S, Park S, Choo J (2005) Complete sequence analysis of the mitochondrial genome in the earthworm, *Perionyx excavatus*. Integrative Biosciences 9: A705.

[B30] Kimura M (1980) A simple method for estimating evolutionary rates of base substitutions through comparative studies of nucleotide sequences. Journal of Molecular Evolution 16: 111–120. 10.1007/bf01731581 7463489

[B31] Koo J, Hong Y (2023) The complete mitochondrial genome of the Korean endemic earthworm *Amynthas deogyusanensis* (Clitellata: Megascolecidae). Mitochondrial DNA Part B 8: 107–109. 10.1080/23802359.2022.2161839 PMC9833400 36643809

[B32] Liu M, Li J, Zhang Y, Aspe NM, Wu D, Zhao H, Zheng G (2025a) A new species of the *Drawida ghilarovi* species complex (Oligochaeta, Moniligastridae) in Changbai Mountain, Northeast China. Zoosystematics and Evolution 101: 627–641. 10.3897/zse.101.146587

[B33] Liu M, Miao P, Liu Z, Aspe NM, Zhang Y, Zhao H (2025b) Two new species of the *Drawida japonica* species complex (Oligochaeta, Moniligastridae) from East Asia delimited by integrative taxonomic methods. ZooKeys 1264: 377–402. 10.3897/zookeys.1264.170881 PMC12743248 41458142

[B34] Meng G, Li Y, Yang C, Liu S (2019) MitoZ: a toolkit for animal mitochondrial genome assembly, annotation and visualization. Nucleic Acids Research 47: e63. 10.1093/nar/gkz173 PMC6582343 30864657

[B35] Michaelsen W (1928) Miscellanea oligochaetologiea. Arkiv för Zoologi 20: 1–15.

[B36] Michaelsen W (1934) Oligochaeten von Franz Ösisch-Indochina. Archives de Zoologie Expérimentale et Générale 76: 493–546.

[B37] Misirlioğlu M, Reynolds JW, Stojanović M, Trakić T, Sekulić J, James SW, Csuzdi C, Decaëns T, Lapied E, Phillips HRP, Cameron EK, Brown GG (2023) Earthworms (Clitellata, Megadrili) of the world: an updated checklist of valid species and families, with notes on their distribution. Zootaxa 5255(1): 417–438. 10.11646/zootaxa.5255.1.33 37045245

[B38] Nguyen TT, Lam DH, Nguyen AD (2021) Notes on the earthworm species, *Metaphire anomala* (Michaelsen, 1907) (Clitellata, Megascolecidae) in Southern Vietnam, with descriptions of two new species. European Journal of Taxonomy 746: 94–111. 10.5852/ejt.2021.746.1321

[B39] Nguyen TT, Ly VV, Lam DH, Nguyen TV, Nguyen AD (2022) A New Earthworm Species of the Genus *Metaphire* Sims & Easton, 1972 (Oligochaeta: Megascolecidae) from Southern Vietnam. Tropical Natural History 22(1): 72–84. 10.58837/tnh.22.1.256982

[B40] Pérez-Losada M, Bloch R, Breinholt JW, Pfenninger M, Domínguez J (2012) Taxonomic assessment of Lumbricidae (Oligochaeta) earthworm genera using DNA barcodes. European Journal of Soil Biology 48: 41–47. 10.1016/j.ejsobi.2011.10.003

[B41] Pérez-Losada M, Eiroa J, Mato S, Domínguez J (2005) Phylogenetic species delimitation of the earthworms *Eisenia fetida* (Savigny, 1826) and *Eisenia andrei* Bouché, 1972 (Oligochaeta, Lumbricidae) based on mitochondrial and nuclear DNA sequences. Pedobiologia 49: 317–324. 10.1016/j.pedobi.2005.02.004

[B42] Phan QT, Lam DH, Tran MT, Pham QV, Nguyen AD (2026) Two new earthworm species of the genus *Metaphire* Sims & Easton, 1972 (Annelida: Oligochaeta: Megascolecidae) from Highlands of Vietnam. Zootaxa 5748(1): 101–112. 10.11646/zootaxa.5748.1.5 42408314

[B43] Pop AA, Wink M, Pop VV (2003) Use of 18S, 16S rDNA and cytochrome c oxidase sequences in earthworm taxonomy (Oligochaeta, Lumbricidae). Pedobiologia 47: 428–433. 10.1078/0031-4056-00208

[B44] Porco D, Decaëns T, Deharveng L, James SW, Skarżyński D, Erséus C, Butt KR, Richard B, Hebert PDN (2012) Biological invasions in soil: DNA barcoding as a monitoring tool in a multiple taxa survey targeting European earthworms and springtails in North America. Biological Invasions 15: 899–910. 10.1007/s10530-012-0338-2

[B45] Puillandre N, Brouillet S, Achaz G (2021) ASAP: assemble species by automatic partitioning. Molecular Ecology Resources 21: 609–620. 10.1111/1755-0998.13281 33058550

[B46] Qiu J, Bouché MB (1998) Révision des taxons supraspécifiques de Lumbricoidea. Documents Pédozoologiques et Intégrologiques 3: 179–216. 10.13140/RG.2.2.24269.49127

[B47] Qiu J, Zhong Y (1993) Notes on a new species and a new subspecies of the genus *Metaphire* from Guizhou Province, China (Haplotaxida: Megascolecidae). Guizhou Science 11: 38–44.

[B48] Rambaut A (2022) Figtree 1.4.4. http://tree.bio.ed.ac.uk/software/figtree/ [accessed on 1 July 2022]

[B49] Rambaut A, Drummond AJ, Xie D, Baele G, Suchard MA (2018) Posterior Summarization in Bayesian Phylogenetics Using Tracer 1.7. Systematic Biology 67: 901–904. 10.1093/sysbio/syy032 PMC6101584 29718447

[B50] Ratmanee C, Anuwat A, Teerapong S, Ueangfa B, Somsak P (2023) Four new terrestrial earthworm species from the northeast Thailand (Oligochaeta, Megascolecidae). ZooKeys 1176: 195–219. 10.3897/zookeys.1176.106517 PMC10477909 37675339

[B51] Ren J, Liu Y, Zhang S, Zheng C, Tan Y, Peng C, Gao J, Hou F (2025) Three New Earthworm Species of the Genera *Amynthas* and *Metaphire* (Oligochaeta: Megascolecidae) From Leshan, China. Zoological Society 42: 206–218 10.2108/zs240010 40184199

[B52] Ronquist F, Teslenko M, van der Mark P, Ayres DL, Darling A, Hohna S, Larget B, Liu L, Suchard MA, Huelsenbeck JP (2012) MrBayes 3.2: efficient Bayesian phylogenetic inference and model choice across a large model space. Systematic Biology 61: 539–542. 10.1093/sysbio/sys029 PMC3329765 22357727

[B53] Rougerie R, Decaëns T, Deharveng L, Porco D, James SW, Chang C-H, Richard B, Potapov M, Suhardjono Y, Hebert PDN (2009) DNA barcodes for soil animal taxonomy. Pesquisa Agropecuária Brasileira 44: 789–802. 10.1590/s0100-204x2009000800002

[B54] Sato C, Nendai N, Nagata N, Okuzaki Y, Ikeda H, Minamiya Y, Sota T (2023) Origin and diversification of pheretimoid megascolecid earthworms in the Japanese Archipelago as revealed by mitogenomic phylogenetics. Molecular Phylogenetics and Evolution 182: 107735. 10.1016/j.ympev.2023.107735 36805472

[B55] Shen H, Tsai SC, Tsai CF (2005) Occurrence of the Earthworms *Pontodrilus litoralis* (Grube, 1855), *Metaphire houlleti* (Perrier, 1872), and *Eiseniella tetraedra* (Savigny, 1826) from Taiwan. Taiwania 50: 11–21. 10.6165/tai.2005.50(1).11

[B56] Shukla R, Soni J, Kumar A, Pandey R (2024) Uncovering the diversity of pathogenic invaders: insights into protozoa, fungi, and worm infections. Frontiers in Microbiology 15: 1374438. 10.3389/fmicb.2024.1374438 PMC11003270 38596382

[B57] Sims RW, Easton EG (1972) A numerical revision of the earthworm genus *Pheretima* auct. (Megascolecidae, Oligochaeta) with the recognition of new genera and an appendix on the earthworms collected by the Royal Society North Borneo Expedition. Biological Journal of the Linnaean Society 4: 169–268. 10.1111/j.1095-8312.1972.tb00694.x

[B58] Stamatakis A (2014) RAxML version 8: a tool for phylogenetic analysis and post-analysis of large phylogenies. Bioinformatics 30: 1312–1313. 10.1093/bioinformatics/btu033 PMC3998144 24451623

[B59] Sun J (2013) Taxonomy and Molecular Phylogeny of *Amynthas* earthworms from China. Doctoral dissertation, Shanghai Jiao Tong University. [In Chinese with English abstract]

[B60] Sun J, Jiang J, Scott B, Qiu J, Feng H (2018) Four new *Amynthas* and *Metaphire* earthworm species from nine provinces in southern China. Zootaxa 4496: 287–301. 10.11646/zootaxa.4496.1.24 30313704

[B61] Tamura K, Peterson D, Peterson N, Stecher G, Nei M, Kumar S (2011) MEGA5: molecular evolutionary genetics analysis using maximum likelihood, evolutionary distance, and maximum parsimony methods. Molecular Biology and Evolution 28: 2731–2739. 10.1093/molbev/msr121 PMC3203626 21546353

[B62] Tan T, Zhong Y (1986) A new species of the genus *Metaphire* from Hunan (Oligochaeta: Megascolecidae). Acta Zootaxonomica Sinica 11: 144–147.

[B63] Tan T, Zhong Y (1987) Two new species of the genus *Metaphire* from Hunan Province (Oligochaeta: Megascolecidae). Acta Zootaxonomica Sinica 12: 128–132.

[B64] Thai TB, Do VN, Huyhn TKH (1992) New species of earthworm of genus *Pheretima* Kinberg, 1867 (Megascolecidae-Oligochaeta) belonging the bank of streams Xuan Nha Moc Chau (Son La Province) and Dac No, Dac Ken (Dac Lac Province). Tap chi Sinh hoc 14(4): 1–3.

[B65] Thai TB, Le VT (1993) Two new earthworms of the genus *Pheretima* Kinberg, 1897 (Megascolecidae-Oligochaeta) from Catba National Park (Cat Ba Island, Hai Phong). Tap chi Sinh hoc 15(4): 33–35.

[B66] Tiwari N, Shilpi K, James SW, Gupta N, Yadav S (2025) Three Novel Species of Earthworms of Genus *Metaphire* Sims and Easton, 1972 from Manipur, India. Zootaxa 5589: 166–189. 10.11646/zootaxa.5589.1.14 40173784

[B67] Wu C (2008) Landform environment and its formation in North China. Science Press, Beijing, 64–66.

[B68] Xu Q, Xiao N (2011) Terrestrial earthworms (Opisthopora: Oligochaeta) of China. China Agriculture Press, Beijing, 66–269.

[B69] Yu X, Yang H, Liu J, Qi Y, Sun L, Tian X (2022) A strategy for a high enrichment of insect mitochondrial DNA for mitogenomic analysis. Gene 808: 145986. 10.1016/j.gene.2021.145986 34600050

[B70] Zhang L, Jiang J, Dong Y, Qiu J (2015) Complete mitochondrial genome of four pheretimoid earthworms (Clitellata: Oligochaeta) and their phylogenetic reconstruction. Gene 574: 308–316. 10.1016/j.gene.2015.08.020 26291739

[B71] Zhang L, Jiang J, Dong Y, Qiu J (2016a) Complete mitochondrial genome of a Pheretimoid earthworm *Metaphire vulgaris* (Oligochaeta: Megascolecidae). Mitochondrial DNA Part A 27: 297–298. 10.3109/19401736.2014.892085 24617491

[B72] Zhang L, Jiang J, Dong Y, Qiu J (2016b) Complete mitochondrial genome of an *Amynthas* earthworm, *Amynthas aspergillus* (Oligochaeta: Megascolecidae). Mitochondrial DNA Part A 27: 1876–1877. 10.3109/19401736.2014.971267 25329289

[B73] Zhang L, Sechi P, Yuan M, Jiang J, Dong Y, Qiu J (2016c) Fifteen new earthworm mitogenomes shed new light on phylogeny within the *Pheretima* complex. Scientific Reports 6: 20096. 10.1038/srep20096 PMC4735579 26833286

[B74] Zhang Y, Bi Y, Li G, Shen L (2017) Relationship between earthworm diversity and soil environment in Hebei area. Journal of China Agricultural University 22: 60–68. 10.11841/j.issn.1007-4333.2017.03.08

[B75] Zhao H, Fan S, Aspe NM, Feng L, Zhang Y (2022) Characterization of 15 Earthworm Mitogenomes from Northeast China and Its Phylogenetic Implication (Oligochaeta: Lumbricidae, Moniligastridae). Diversity 14: 714. 10.3390/d14090714

[B76] Zhao H, Han ACN, Liu M, Zhang Y, Aspe NM, Miao P, Wu D (2025) A new pheretimoid earthworm of the genus *Metaphire* Sims & Easton, 1972 (Oligochaeta, Megascolecidae) from northeastern China with data from the mitochondrial genome. Zoosystematics and Evolution 101: 81–89. 10.3897/zse.101.136027

[B77] Zhao Q (2015) Taxonomy, phylogeny and paleogeography of pheretimoid earthworm species in Hainan island (China). Doctoral dissertation, Shanghai Jiao Tong University. [In Chinese with English abstract]

[B78] Zhong Y, Xu X, Wang D (1984) On a new species of the earthworm genus *Pheretima* and its reproductive organ polymorphism. Acta Zootaxonomica Sinica 9: 356–360.

